# Robustness of Entanglement for Dicke-W and Greenberger-Horne-Zeilinger Mixed States

**DOI:** 10.3390/e26090804

**Published:** 2024-09-21

**Authors:** Ling-Hui Zhu, Zhen Zhu, Guo-Lin Lv, Chong-Qiang Ye, Xiao-Yu Chen

**Affiliations:** 1 School of Information and Electrical Engineering, Hangzhou City University, Hangzhou 310015, China; 2230201010@stu.hzcu.edu.cn (L.-H.Z.); 221122030296@zjut.edu.cn (Z.Z.); 22231168@zju.edu.cn (G.-L.L.); chongqiangye@aliyun.com (C.-Q.Y.); 2 College of Information Engineering, Zhejiang University of Technology, Hangzhou 310014, China; 3 College of Information Science & Electronic Engineering, Zhejiang University, Hangzhou 310027, China

**Keywords:** quantum entanglement, robustness of entanglement, Dicke state, W state, GHZ state

## Abstract

Quantum entanglement is a fundamental characteristic of quantum mechanics, and understanding the robustness of entanglement across different mixed states is crucial for comprehending the entanglement properties of general quantum states. In this paper, the robustness of entanglement of Dicke–W and Greenberger–Horne–Zeilinger (GHZ) mixed states under different mixing ratios is calculated using the entanglement witness method. The robustnesses of entanglement of Dicke–W and GHZ mixed states are different when the probability ratio of Dicke to W is greater than $\frac{3}{2}$ and less than $\frac{3}{2}$. For the probability of Dicke and W states greater than or equal to $\frac{3}{2}$, we study the robustness of entanglement of Dicke and GHZ mixed states and analyze and calculate their upper and lower bounds. For the probability of Dicke and W states less than $\frac{3}{2}$, we take the equal probability ratio of Dicke and W states as an example and calculate and analyze the upper and lower bounds of their robustness of entanglement in detail.

## 1. Introduction

In quantum information theory, quantum entanglement [1] plays a fundamental role as a resource in quantum communication between two parties separated by macroscopic distances [2]. A state is called separable if it can be written as the probability mixture of product states [3]; otherwise, it is entangled. However, detecting entanglement remains an NP-hard problem.

To address this challenge, considerable work has been conducted on developing criteria for judging entanglement or separability. The most widely used is the positive partial transpose (PPT) criterion [4,5] since it provides a complete characterization of entanglement for two-qubit systems, and studies have shown that violating PPT conditions can be used to quantify entanglement [6,7]. The computable cross norm or realignment criterion (CCNR criterion) [8,9] is also a strong criterion that can be considered as a supplement to the PPT criterion. Entanglement witnesses are Hermitian operators for detecting entanglement [10,11,12,13] and are a necessary entanglement criterion in terms of directly measurable observables. It is a very useful tool for the analysis of entanglement in experiments, but it still cannot solve all entanglement problems because the construction of witnesses is still difficult. The range criterion [14] is used for determining the separability of mixed states based on the range of the density matrix. This criterion is generally used when the PPT criterion fails. Compared with the PPT criterion, the range criterion has wider applicability and can detect more quantum entanglement states. However, the range criterion also has limitations and does not apply to situations where quantum states are disturbed by noise.

The theory of entanglement has been gradually improved and has become a core theme of quantum information science. At the same time, the development of quantum entanglement has also driven the development of many fields such as quantum dense coding [15,16], quantum teleportation [17,18], entanglement purification [19], quantum error correction [2,20], and quantum memory [21].

Entanglement in multipartite systems is a key resource for quantum information and communication protocols [1,22]. In experiments, different multipartite entangled states have been prepared [23,24,25]. Determining whether the state produced in the experiment is a multipartite (partial) entangled state has become a highly relevant topic in quantum information theory [26]. The Greenberger–Horne–Zeilinger (GHZ) state is also called the maximally entangled state. Due to its maximum entanglement characteristics and measurement accuracy approaching the Heisenberg limit [27], it has considerable application prospects in the fields of quantum information, quantum communication, and precision measurement. At the same time, due to the special properties of the GHZ state, it can be used in multipartite secret sharing protocols. In addition, when the entangled GHZ state is used as a resource, the photonic architecture of measurement-based quantum computing becomes more efficient [28]. Chen et al. [29] proposed a road map for finding the separability criteria of multipartite entangled states and derived a set of tripartite separability criteria for the four-qubit GHZ diagonal states. The Dicke state [30], proposed by R.H. Dicke in 1954, is of great significance for studying the properties of multi-particle quantum entanglement and building multi-user quantum networks because its entanglement is robust to particle loss and is attractive in practical applications such as multi-party quantum networks [31] and quantum metrology. The W state corresponds to the Dicke state $|Dicke_{3,1}\rangle$, and therefore, examples of a Dicke state [6] and W states have already been prepared in many experiments [32,33,34]. Chen et al. [35] demonstrated an entanglement criterion for any four-qubit state which is necessary and sufficient for the generalized noisy four-qubit Dicke states. Carvalho [36] showed that there is a clear scaling of the entanglement decay rates for the GHZ and W states for various environments. Zhang [37] found that the scar state encompasses both GHZ and W states, which provides the possibility of thermal-free quantum information processing in finite-sized quantum spin clusters. Chen et al. [38] showed that for a mixture of a four-qubit GHZ state with a Dicke state and white noise, there exists a new Wootters formula. Therefore, the measurement and detection of the robustness of entanglement of Dicke–W and GHZ mixed states is of great significance for quantum information applications and the preparation of related quantum states.

In this paper, we give the numerical analysis and theoretical interpretation of the robustness of entanglement of the full separability of Dicke–W and GHZ mixed states under two different mixing ratios. By plotting the figure of the robustness of entanglement, we find two different types of figures and then analyze the mixing ratio of the boundary between the two types.

The paper is organized as follows: In Section 2, we introduce the concept of robustness of entanglement and some concepts involved in the paper. Section 3 elaborates on the numerical and theoretical analysis methods for the robustness of entanglement in the fully separable case of Dicke–GHZ mixed states. Section 4 shows the results of the robustness of entanglement for Dicke–W and GHZ when the mixing ratio of Dicke–W is $45^{\circ }$ in the case of full separability and also gives the results for the upper and lower bounds. Section 5 gives the boundary of the mixing ratio. Section 6 is the conclusions.

## 2. Preliminary

A multipartite state ρ is separable when it can be written as

(1) $$\rho =\sum_{i}p_{i}\rho_{i}^{A_{1}}⊗\rho_{i}^{A_{2}}\cdots ⊗\rho_{i}^{A_{N}},$$

where $\rho_{i}^{A_{j}}$ is the state of $A_{j}$, and $p_{i}$ is the probability distribution.

In our work, we explore the robustness of entanglement of Dicke–W and GHZ mixed states in different ratios. The density matrix for a mixed state consisting of Dicke–W and GHZ is given by

(2) $$\rho =p_{1}|GHZ_{4}\rangle \langle \cdot |+\left(1-p_{1}\right)[p_{2}|Dicke_{4,2}\rangle \langle \cdot |+\left(1-p_{2}\right)|W_{4}\rangle \langle \cdot |],$$

where $\left\{p_{1},\left(1-p_{1}\right)p_{2},\left(1-p_{1}\right)\left(1-p_{2}\right)\right\}$ denote the probability distributions, and we abbreviate |χ〉〈χ|=|χ〉〈·|. $|GHZ_{4}\rangle =\frac{1}{\sqrt{2}}(|0000\rangle +\left|1111\rangle \right)$, $|Dicke_{4,2}\rangle =\frac{1}{\sqrt{6}}\sum_{\left|k\right|=2}|k\rangle$, $|W_{4}\rangle =\frac{1}{2}\sum_{\left|k\right|=1}|k\rangle$, with $k\in {\left\{0,1\right\}}^{⊗4}$ being a binary vector and $\left|k\right|$ being the Hamming weight of k. We denote $\tan\Theta =\frac{p_{1}}{1-p_{1}}$ and $\tan\Phi =\frac{p_{2}}{1-p_{2}}$, and we call Φ the mixing angle of Dicke and W states. We will omit the subscripts of GHZ, Dicke, and W for simplicity.

The definition of robustness is given by Lami et al. [39] for any state ρ and is expressed as follows:

(3) $$R_{F}\left(\omega \right):=\inf\left\{1+\lambda \left|\;\frac{\omega +\lambda \tau }{1+\lambda }\in F\right.,\tau \in D\left(H\right)\right\},$$

where τ∈D(H) are noise states defined on Hilbert space H, D(H) is the density matrix in it, and the separable state set F is generally assumed to be convex and closed. Equation (3) leads to the upper bound of the robustness of entanglement, and for any states ρ, ω∈D(H) it holds that

(4) $${\underline{R}}_{F}\left(\rho \right)\geq \frac{\langle \rho ,\omega \rangle }{\sup_{\sigma \in F}\langle \sigma ,\omega \rangle },$$

where σ is a separable state and $\underline{R}$ represents the lower bound.

The minimum quantity of noise state 1+λ is the upper bound of robustness, and the free state reaches the optimum, denoted as σ. We let $\tilde{\rho }=\frac{\rho }{1+\lambda }$, which can be expressed as

(5) $$\tilde{\rho }=\frac{p_{1}}{1+\lambda }|GHZ\rangle \langle \cdot |+\frac{1-p_{1}}{1+\lambda }[p_{2}|Dicke\rangle \langle \cdot |+\left(1-p_{2}\right)|W\rangle \langle \cdot |].$$

We denote $g=\frac{p_{1}}{1+\lambda }$, $d=\frac{1-p_{1}}{1+\lambda }p_{2}$ and $w=\frac{1-p_{1}}{1+\lambda }\left(1-p_{2}\right)$. Then *g*, *d*, and *w* represent the coefficients of |GHZ〉 state, |Dicke〉 state, and |W〉 state in σ, respectively. For ease of presentation, we use $d_{w}=\sqrt{d^{2}+w^{2}}$ as the figure’s horizontal axis. It can be seen that $g+\frac{d_{w}}{\sqrt{p_{2}^{2}+{\left(1-p_{2}\right)}^{2}}}=\frac{1}{1+\lambda }=\frac{1}{R}$ and $\frac{g}{d_{w}}=$$\frac{p_{1}}{1-p_{1}}$=tanΘ. Thus, the relationship between $d_{w}$, *g*, and *R* will be displayed in the figure shown later by Θ.

## 3. Robustness of Fully Separable Four-Qubit Dicke–GHZ Mixed States $\left(\Phi =90^{\circ }\right)$

In this section, $\Phi =90^{\circ }$, so the density matrix $\rho_{1}$ in Equation (2) will be given by

(6) $$\rho_{1}=p|GHZ\rangle \langle GHZ|+\left(1-p\right)|Dicke\rangle \langle Dicke|,$$

where *p* and 1−p denote the probability distributions. A density matrix ρ can be expressed using the 2×2 identity matrix $\sigma_{0}$ and Pauli matrices $\sigma_{1}$, $\sigma_{2}$, and $\sigma_{3}$ as follows:

(7) $$\rho =\frac{1}{2^{4}}\sum_{i,j,j,l=0}^{3}R_{ijkl}\sigma_{i}⊗\sigma_{j}⊗\sigma_{k}⊗\sigma_{l},$$

where $R_{ijkl}=Tr\left(\rho_{s}\right)\sigma_{i}⊗\sigma_{j}⊗\sigma_{k}⊗\sigma_{l}$. The mixed states $\rho_{1}$ in Equation (6) can be expressed as

(8) $$\begin{array}{cc} \rho_{1}=\frac{1}{16}[ & IIII+R_{1}IIIZ+R_{2}IIZI+R_{3}IZII+R_{4}ZIII\\ + & R_{5}IZZZ_{p}+R_{6}\left(IIXX_{p}+IIYY_{p}\right)+R_{7}(XXZZ_{p}\\ + & YYZZ_{p})+R_{8}IXXZ_{p}+M_{9}IYYZ_{p}+R_{10}ZZZZ\\ + & R_{11}IIZZ_{p}+R_{12}XXYY_{p}+R_{13}XXXX+M_{14}YYYY], \end{array}$$

where the subscript *p* denotes the summation over all permutations of Pauli matrices, and I, X, Y, and Z denote the Pauli matrices $\sigma_{0}$, $\sigma_{1}$, $\sigma_{2}$, and $\sigma_{3}$, respectively. The characteristic vector is expressed in the form of

(9) $$\begin{array}{c} R=(R_{1},R_{2},R_{3},R_{4},4R_{5},12R_{6},12R_{7},12R_{8},12R_{9},R_{10},6R_{11},6R_{12},R_{13},R_{14}{)}^{T}. \end{array}$$

For mixed states of Dicke and GHZ, the witness ω of Equation (4) can be expressed in terms of the parameters $M_{i}$ and the tensor product of Pauli matrices as follows:

(10) $$\omega =M_{0}IIII+\hat{M^{\prime }},$$

and the trace-free matrix $\hat{M^{\prime }}$ can be expressed as

(11) $$\begin{array}{cc} \hat{M^{\prime }}= & M_{1}IIIZ+M_{2}IIZI+M_{3}IZII+M_{4}ZIII+M_{5}IZZZ_{p}\\ + & M_{6}\left(IIXX_{p}+IIYY_{p}\right)+M_{7}\left(XXZZ_{p}+YYZZ_{p}\right)\\ + & M_{8}IXXZ_{p}+M_{9}IYYZ_{p}+M_{10}ZZZZ+M_{11}IIZZ_{p}\\ + & M_{12}XXYY_{p}+M_{13}XXXX+M_{14}YYYY, \end{array}$$

where the subscript *p* denotes the summation over all permutations of Pauli matrices.

To find the maximum value of the denominator in Equation (4), we transform it into finding the maximum of $\langle \psi_{s}|\omega |\psi_{s}\rangle$ and $|\psi_{s}\rangle =|\psi_{1}\rangle |\psi_{2}\rangle |\psi_{3}\rangle |\psi_{4}\rangle$. The product state $|\psi_{k}\rangle$ can be expressed in the Bloch representation as $|\psi_{k}\rangle =\cos\frac{\theta_{k}}{2}|0\rangle +\sin\frac{\theta_{k}}{2}e^{i\varphi_{k}}|1\rangle$, (k=1,2,3,4), where θ is the polar angle, and φ is the azimuthal angle. The density matrix of $\rho_{k}=|\psi_{k}\rangle \langle \psi_{k}|$ can be represented by Pauli matrices as

(12) $$\rho_{k}=\frac{1}{2}\left(I+x_{k}\sigma_{1}+y_{k}\sigma_{2}+z_{k}\sigma_{3}\right),$$

where $x_{k}=\sin\theta_{k}\cos\varphi_{k}$, $y_{k}=\sin\theta_{k}\sin\varphi_{k}$, $z_{k}=\cos\theta_{k}$, (k=1,2,3,4). $Tr(\hat{M}|\psi_{s}\rangle \langle \psi_{s}|)=M_{0}+\Lambda$, and $\Lambda =\Lambda_{1}+\Lambda_{2}x_{4}+\Lambda_{3}y_{4}+\Lambda_{4}z_{4}$, then

(13) $$\Lambda =\underset{\theta_{1}\theta_{2}\theta_{3}\varphi_{1}\varphi_{2}\varphi_{3}}{max}\Lambda_{1}+\sqrt[{\;}]{\Lambda_{2}^{2}+\Lambda_{3}^{2}+\Lambda_{4}^{2}},$$

where $\Lambda_{i}\left(i=1,2,3,4\right)$ is a function with variables $M_{j},x_{k},y_{k},z_{k}$. More details are given in the Appendix A. The numerator of Equation (4) can be expressed as

(14) $$\langle \rho ,\omega \rangle =M_{0}+M\cdot \frac{R}{\cos\Theta +\sin\Theta }.$$

The denominator of Equation (4) can be expressed as

(15) $$\langle \sigma ,\omega \rangle =M_{0}+\Lambda ,$$

where the vector $M=(M_{1},\cdots ,M_{14})$. Given a Dicke–GHZ state ρ, we adjust the parameter vector M to achieve the minimum value of L,

(16) $$L=\frac{M_{0}+\Lambda }{M_{0}\left(\cos\Theta +\sin\Theta \right)+M\cdot R}.$$

**Figure 1:**
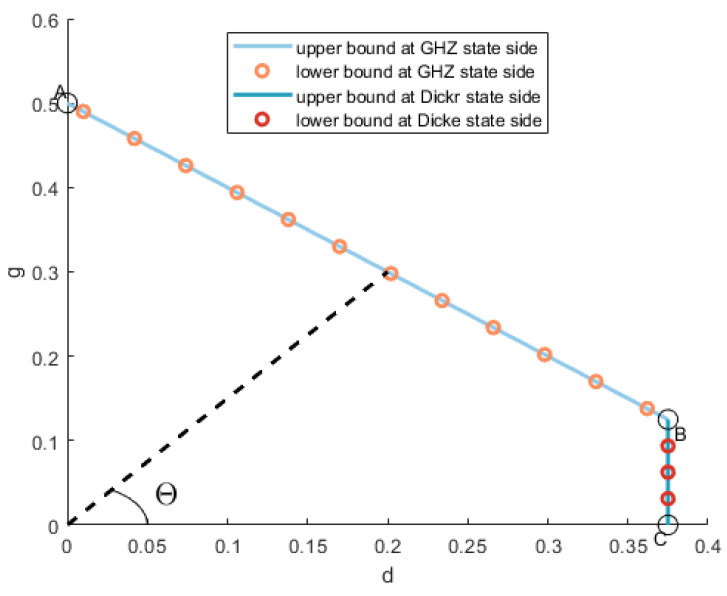
The robustness of entanglement for four-qubit Dicke–GHZ mixed states. The curve can be divided into two parts, and each part consists of a lower bound and an upper bound. The angle between the horizontal axis of the coordinate and the dotted line is called Θ, and it satisfies $\tan\Theta =\frac{p}{1-p}$. Points *A*, *B*, and *C* divide the curve into two parts. Line $\bar{AB}$ corresponds to $\Theta \in \left[18.4349^{\circ },90^{\circ }\right]$, while line $\bar{BC}$ corresponds to $\Theta \in \left[0^{\circ },18.4349^{\circ }\right]$. The coordinates of points *A*, *B*, and *C* are $\left(0,\frac{1}{2}\right)$, $\left(\frac{3}{8},\frac{1}{8}\right)$, and $\left(\frac{3}{8},0\right)$, respectively.

The robustness of entanglement of four-qubit Dicke–GHZ mixed states is described by the curve in Figure 1.

### 3.1. Segment AB

For Dicke–GHZ mixed states at the GHZ state side, numerical calculation suggests

(17) $$\begin{array}{c} M_{1}=M_{2}=M_{3}=M_{4}=M_{5}=M_{8}=M_{9}=0\\ M_{13}=M_{14}. \end{array}$$

By analyzing and calculating, we get the relationship among $M_{i}$:

(18) $$\begin{array}{cc} \; & M_{10}=6\left(M_{6}-M_{11}\right)+M_{13}\\ \; & M_{12}=M_{7}+M_{11}-M_{6}. \end{array}$$

and we find that when $\theta =90^{\circ }$ and $\varphi =\frac{k\pi }{2}$ where k=0,1,2,3, the Λ of Equation (13) will achieve the same maximum value:

(19) $$\Lambda =6M_{6}+M_{13}.$$

The $M_{0}$ in Equation (15) can be expressed as

(20) $$M_{0}=6\left(-M_{7}-M_{11}\right)+M_{13},$$

and the rest of Equation (15) can be expressed as

(21) $$\begin{array}{cc} M\cdot R= & M_{6}8\cos\Theta +M_{7}\left(-8\cos\Theta \right)+M_{10}\left(\cos\Theta +\sin\Theta \right)\\ \; & +M_{11}\left(-2\cos\Theta +6\sin\Theta \right)+M_{12}\left(2\cos\Theta -6\sin\Theta \right)\\ \; & +2M_{13}\left(\cos\Theta +\sin\Theta \right). \end{array}$$

Combining the analysis among parameters and Equation (16), we get $L=\frac{1}{2(\cos\Theta +\sin\Theta )}$. Comparing Equation (4) and Equation (16), we can easily get $L=\frac{1}{R(\cos\Theta +\sin\Theta )}$, and by converting it to the Descartes coordinate system, we get

(22) $$g+d=\frac{1}{2},$$

where $g=\frac{\sin\Theta }{R(\sin\Theta +\cos\Theta )}$ and $d=\frac{\cos\Theta }{R(\sin\Theta +\cos\Theta )}$. The criterion is shown in Figure 1, with the blue line indicating Line AB and accounting well for the numerical necessary criterion when $\Theta \in [\Theta_{1},90^{\circ }]$ with the angle $\Theta_{1}=\tan^{-1}\frac{1}{3}\approx 18.4349^{\circ }.$

Next, we will present the upper bound of this segment. A product state $|\psi_{s}\rangle =(c|0\rangle +se^{i\varphi }{\left|1\rangle \right)}^{⊗4}$ can be represented as

(23) $$\begin{array}{cc} |\psi_{s}\rangle = & c^{4}|0000\rangle +s^{4}e^{i4\varphi }|1111\rangle \\ & +c^{3}se^{i\varphi }(|0001\rangle +|0010\rangle +|0100\rangle +\left|1000\rangle \right)\\ & +c^{2}s^{2}e^{i2\varphi }(|0011\rangle +|0101\rangle +|0110\rangle +|1001\rangle +|1010\rangle +\left|1100\rangle \right)\\ & +cs^{3}e^{i3\varphi }(|0111\rangle +|1011\rangle +|1101\rangle +|1110\rangle ), \end{array}$$

where $c=\cos\frac{\theta }{2}$, $s=\sin\frac{\theta }{2}$. Let $\varphi =\frac{k\pi }{2}$; we define the separable state $\sigma =\frac{1}{4}\sum_{k=0}^{3}|\psi_{s}\rangle \langle \psi_{s}|$. The separable state σ can be expressed as

(24) $$\begin{array}{cc} \sigma = & c^{8}\left|0000\rangle \langle 0000\right|+s^{8}\left|1111\rangle \langle 1111\right|\\ & +c^{4}s^{4}(|0000\rangle \langle 1111|+|1111\rangle \langle 0000|)\\ & +4c^{6}s^{2}\left|W\rangle \langle W\right|\\ & +6c^{4}s^{4}\left|Dicke\rangle \langle Dicke\right|\\ & +4c^{2}s^{6}|\bar{W}\rangle \langle \bar{W}|, \end{array}$$

where $|\bar{W}\rangle =\frac{1}{2}(|0111\rangle +|1011\rangle +|1101\rangle +\left|1110\rangle \right)$.

When $\Phi =90^{\circ }$, ρ is a Dicke–GHZ mixed state, $\theta =\frac{\pi }{2}$, $\varphi =\frac{k\pi }{2}$, k=0,1,2,3; thus, the separable state $\sigma_{B}$ for point *B* in Figure 1 can be expressed as

(25) $$\begin{array}{cc} \sigma_{B}= & \frac{1}{8}\left|GHZ\rangle \langle GHZ\right|+\frac{3}{8}\left|Dicke\rangle \langle Dicke\right|+\frac{1}{4}\left|W\rangle \langle W\right|+\frac{1}{4}|\bar{W}\rangle \langle \bar{W}|. \end{array}$$

The point *B* is located at $\left(\frac{3}{8},\frac{1}{8}\right)$ with $\Theta =18.4349^{\circ }$. When $\Theta =90^{\circ }$, the separable state $\sigma_{A}$ of point *A* is

(26) $$\begin{array}{c} \sigma_{A}=\frac{1}{2}(|GHZ\rangle \langle GHZ|+|GHZ_{-}\rangle \langle GHZ_{-}|), \end{array}$$

where $|GHZ_{-}\rangle =\frac{1}{\sqrt{2}}(|0000\rangle -\left|1111\rangle \right)$. Any separable state $\sigma_{AB}$ on segment AB can be expressed as

(27) $$\begin{array}{c} \sigma_{AB}=p\sigma_{A}+\left(1-p\right)\sigma_{B}, \end{array}$$

where *p* and 1−p denote the probability distributions. We have $g=\frac{p}{2}+\frac{1-p}{8}$, $d=\frac{3}{8}\left(1-p\right)$; thus, the expression of segment AB can be represented as

(28) $$\begin{array}{c} g+d=\frac{1}{2}. \end{array}$$

### 3.2. Segment BC

By analyzing and calculating, we get the relationship among $M_{i}$:

(29) $$\begin{array}{c} -M_{10}-6M_{11}=M_{10}-6\left(M_{6}+M_{7}\right)\\ M_{12}=\frac{1}{3}M_{13}. \end{array}$$

and we find that when $\theta =90^{\circ }$ and $\varphi =\frac{k\pi }{2}$ where k=0,1,2,3, the Λ of Equation (13) will achieve the same maximum value:

(30) $$\Lambda =6M_{6}+M_{13}.$$

The $M_{0}$ in Equation (15) can be expressed as

(31) $$M_{0}=M_{10}-6\left(M_{6}+M_{7}\right),$$

and the rest of Equation (15) is equal to that of Equation (21).

Combining the above analysis and conclusions, we get $L=\frac{3}{8}\frac{1}{\cos\Theta }$. According to the relationship between L and *R*, in the Descartes coordinate system, we can easily get

(32) $$d=\frac{3}{8},$$

where $d=\frac{\cos\Theta }{R(\sin\Theta +\cos\Theta )}$.

The criterion is shown in Figure 1, with the blue line representing Line $\bar{BC}$ and accounting for the numerical necessary criterion well when $\Theta \in [0^{\circ },\Theta_{1}]$ with the angle $\Theta_{1}=\tan^{-1}\frac{1}{3}\approx 18.4349^{\circ }$.

Next, we will present the upper bound of this segment. As Θ gradually approaches $0^{\circ }$, the |Dicke〉 state in $\sigma_{B}$ is gradually classified as a noise state. The separable state for point *C* can be represented as

(33) $$\sigma_{C}=\frac{3}{8}\left|Dicke\rangle \langle Dicke\right|+\frac{5}{8}\tau$$

where τ is the noise state. Hence, the coordinate of point *C* can easily be obtained, which is $\left(\frac{3}{8},0\right)$. Segment BC can be expressed as

(34) $$\begin{array}{c} d=\frac{3}{8}. \end{array}$$

## 4. Robustness of Fully Separable Four-Qubit Dicke–W Mixed with GHZ ($\Phi =56.31^{\circ }$)

**Figure 2:**
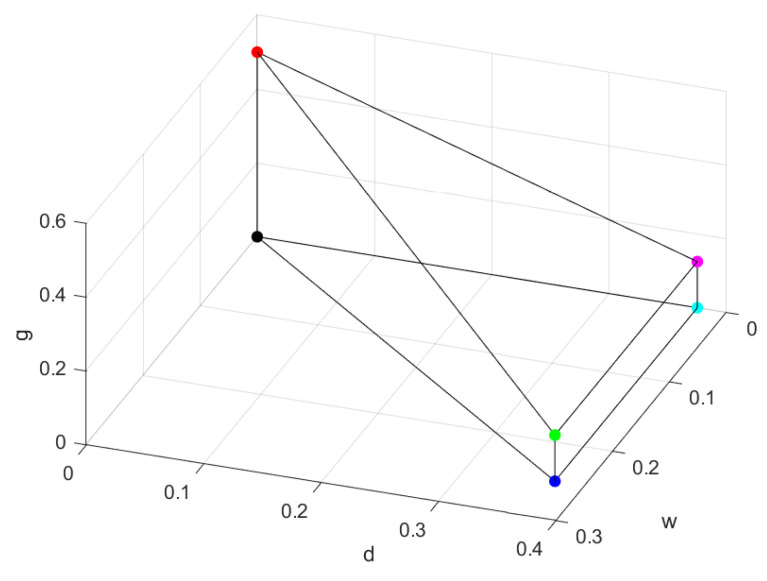
The robustness of fully separable four-qubit Dicke–W and GHZ mixed states. The coordinates of the green point in the figure are ($\frac{1}{4}$, $\frac{3}{8}$, $\frac{1}{8}$).

When ρ is a Dicke–W and GHZ mixed state, the component of the |W〉 state in Equation (24) should be taken into account. However, for $\Phi \in [56.31^{\circ },90^{\circ }]$, the only difference from the result at $\Phi =90^{\circ }$ is that the abscissa is transformed from *d* to $d_{w}$. A schematic representation is depicted in Figure 2.

The reason for this is that when $\theta =\frac{\pi }{2}$, the ratio of the |Dicke〉 to |W〉 components is $\frac{6c^{4}s^{4}}{4c^{6}s^{2}}=\frac{3}{2}$, and $\arctan\frac{3}{2}=56.31^{\circ }$. The green point in Figure 2 located at $\left(w,g,d\right)=(\frac{1}{4},\frac{3}{8},\frac{1}{8}$) represents the unnormalized state $\tilde{\rho }=\frac{1}{8}\left|GHZ\rangle \langle GHZ\right|+\frac{3}{8}\left|Dicke\rangle \langle Dicke\right|+\frac{1}{4}\left|W\rangle \langle W\right|$. Clearly, it is a part of the separable state $\sigma_{B}$ in Equation (25). Thus, the robustness of a state with $\Phi =56.31^{\circ }$ can easily be obtained. When $\Phi <56.31^{\circ }$, due to the insufficient components of the |W〉 state, θ begins to vary. We will show this case in the next section with an example.

## 5. Robustness of Fully Separable Four-Qubit Dicke–W Mixed with GHZ ($\Phi =45^{\circ }$)

In this section, $\Phi =45^{\circ }$, so the density matrix ρ can be expressed as Equation (2). In this case, the entanglement witness for four-qubit Dicke–W and GHZ mixed states is represented using Pauli matrices in the following form:

(35) $$\begin{array}{cc} \omega = & M_{1}IIII+M_{2}IIIZ_{p}+M_{3}IIXX_{p}+M_{4}IIYY_{p}\\ \; & +M_{5}IXXZ_{p}+M_{6}IYYZ_{p}+M_{7}IZZZ_{p}+M_{8}XXZZ_{p}\\ \; & +M_{9}YYZZ_{p}+M_{10}ZZZZ+M_{11}IIZZ_{p}+M_{12}XXXX\\ \; & +M_{13}XXYY_{p}+M_{14}YYYY, \end{array}$$

where the subscript *p* denotes the summation over all permutations of Pauli matrices.

After analysis and calculation, we divide the robustness of the mixed state of four-qubit Dicke–W mixed with GHZ at a 45° ratio into four segments, the specific contents of which are shown in Table 1.

**Figure 3:**
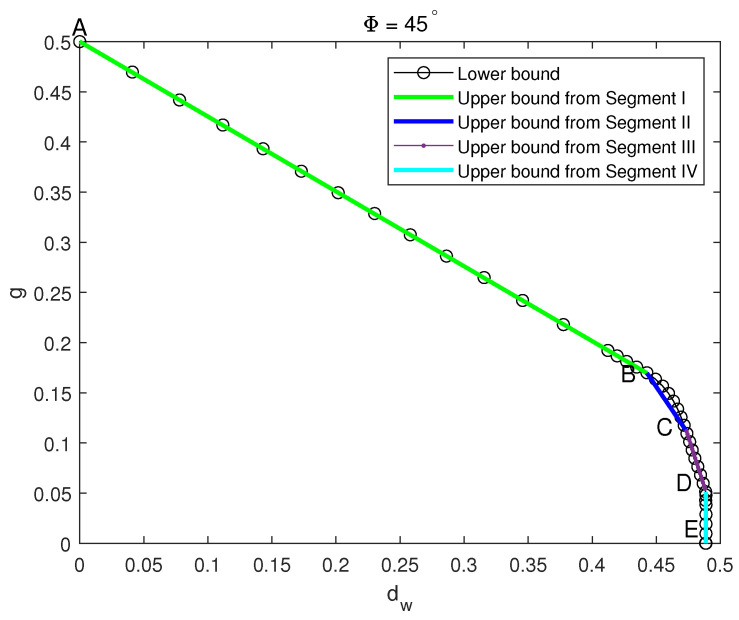
Robustness of entanglement for four-qubit of Dicke–W and GHZ mixed states with a $45^{\circ }$ Dicke–W ratio.

The robustness of entanglement for four-qubit of Dicke–W and GHZ mixed states with a $45^{\circ }$ Dicke–W mixing ratio is shown in Figure 3.

In this section, we employ $\Phi =45^{\circ }$. The upper bound of the robustness is depicted in Figure 3. Based on the characteristics of the figure, several points are labeled as *A*, *B*, *C*, *D*, and *E*. To avoid misunderstanding, the points *A*, *B*, *C*, *D*, and *E* mentioned in this section refer to the points in Figure 3 rather than the points in Figure 1 of Section 3. The analytical solutions for each segment with the deductions are presented in the following text.

### 5.1. Segment I

In this segment, the entanglement witness matrix ω in Equation (4) can be divided into three parts—$\omega_{1}$, $\omega_{2}$, and $\omega_{3}$—which are as follows:

(36) $$\omega =\omega_{1}⊕\omega_{2}⊕\omega_{3},$$

where $\omega_{1}$ represents the witness matrix in the corresponding subspace of the W state, $\omega_{2}$ represents the witness matrix in the corresponding subspace of the Dicke state, and $\omega_{3}$ represents the witness matrix in the corresponding subspace of the GHZ state. In the subspace formed by |0001〉, |0010〉, |0100〉, and |1000〉, $\omega_{1}$ can be represented as

(37) $$\omega_{1}=bI_{4}+aD_{4},$$

where *a* and *b* are positive parameters, $I_{4}$ is a 4×4 identity matrix, and $D_{4}$ is a 4×4 matrix with all elements equal to one. In the subspace formed by |0011〉, |0101〉, |0110〉, |1001〉, |1010〉, and |1100〉, $\omega_{2}$ can be represented as

(38) $$\omega_{2}=dI_{6}+eD_{6}+fG_{6},$$

where *d*, *e*, and *f* are positive parameters, $I_{6}$ is a 6×6 identity matrix, $D_{6}$ is a 6×6 matrix with all elements equal to one, and $G_{6}$ is a 6×6 matrix and can be expressed as follows:

(39) $$\begin{array}{cc} G_{6} & =|0011\rangle \langle 1100|+|0101\rangle \langle 1010|+|0110\rangle \langle 1001|\\ \; & +|1001\rangle \langle 0110|+|1010\rangle \langle 0101|+|1100\rangle \langle 0011|. \end{array}$$

In the subspace formed by |0000〉 and |1111〉, $\omega_{3}$ can be represented as

(40) $$\omega_{3}=hI_{2},$$

where *h* is a positive parameter, and $I_{2}$ is a 2×2 identity matrix.

After completing the construction of the density matrix, we can calculate and analyze the lower bound of the robustness of entanglement through Equation (4). The denominator in Equation (4) can be represented as

(41) $$\langle \psi_{4}\left|\omega \right|\psi_{4}\rangle =hc^{8}+hs^{8}+6\left(d+6e+f\right)c^{4}s^{4}+4\left(4b+a\right)c^{6}s^{2},$$

where $|\psi_{4}{\rangle =|\psi \rangle }^{⊗4}$, $c=\cos\frac{\theta }{2}$, and $s=\sin\frac{\theta }{2}$. According to the analytical calculation shown in Table 1, we have determined that in this part, there are three θ that simultaneously cause Equation (41) to reach the maximum value, one of which is θ=π, and the maximum is *h*. Substituting the maximum value *h* into Equation (41), we can easily obtain

(42) $$c^{8}+s^{8}+\frac{6\left(d+6e+f\right)c^{4}s^{4}+4\left(4b+a\right)c^{6}s^{2}}{h}-1=0.$$

Apart from θ=0 and θ=π, we substitute $\theta =78.4630^{\circ }$ into the formula and can easily get the relationship between other parameters and *h*. The numerator in Equation (4) can be expressed as

(43) $$\langle \rho ,\omega \rangle =\frac{1-p_{1}}{2}\left[p_{2}\left(d+6e+f\right)+\left(1-p_{2}\right)\left(4a+b\right)\right]+2p_{1}h.$$

Combining Equation (4), Equation (43), and the relationship between *h* and the other parameters derived from Equation (42), we can get the lower bound result of robustness of entanglement in this segment:

(44) $$2g+\frac{19}{9\sqrt{2}}d_{w}=1,$$

where $g=\frac{p_{1}}{R}$ and $d_{w}=\left(1-p_{1}\right)\frac{\sqrt{\left(p_{2}^{2}+{\left(1-p_{2}\right)}^{2}\right)}}{R}$. Equation (44) is the expression of Segment I of the lower bound of robustness for the Dicke–W and GHZ mixed states with a Dicke–W mixing ratio of 45°.

Next, we will present the upper bound of this segment. When $\Phi =45^{\circ }$, the coefficients of |W〉 and |Dicke〉 are equal; thus, we have $4c^{6}s^{2}=6c^{4}s^{4}$, $c^{2}=\frac{3}{5}$, and $s^{2}=\frac{2}{5}$. Substituting *c* and *s* back into Equation (24), σ can be expressed as

(45) $$\begin{array}{cc} \sigma = & \frac{81}{625}\left|0000\rangle \langle 0000\right|+\frac{16}{625}\left|1111\rangle \langle 1111\right|\\ & +\frac{36}{625}(|0000\rangle \langle 1111|+|1111\rangle \langle 0000|)\\ & +\frac{216}{625}(|W\rangle \langle W|+|Dicke\rangle \langle Dicke|)\\ & +\frac{96}{625}|\bar{W}\rangle \langle \bar{W}|. \end{array}$$

When $\theta =0^{\circ }$ or $180^{\circ }$, $\sigma_{1}=\left|0000\rangle \langle 0000\right|$ and $\sigma_{2}=\left|1111\rangle \langle 1111\right|$ based on Equation (24). Therefore, the component of |GHZ〉 can be increased by adding $\sigma_{1}$ and $\sigma_{2}$ to σ in Equation (45). Actually, it suffices to add $\sigma_{2}$ in σ. The separable state $\sigma_{B}$ for point *B* can be expressed as

(46) $$\begin{array}{cc} \sigma_{B}= & \frac{625}{690}\left(\sigma +\frac{65}{625}\sigma_{2}\right)\\ = & \frac{39}{230}\left|GHZ\rangle \langle GHZ\right|+\frac{72}{230}(|W\rangle \langle W|+|Dicke\rangle \langle Dicke|)\\ & +\frac{32}{230}|\bar{W}\rangle \langle \bar{W}|+\frac{15}{230}|GHZ_{-}\rangle \langle GHZ_{-}|. \end{array}$$

Point *B* is $\left(\frac{72}{230}\sqrt{2},\frac{39}{230}\right)$ with $\Theta =20.96^{\circ }$. For $\Theta =90^{\circ }$, for example, ρ is a |GHZ〉 pure state, and point *A* is known to be $\left(0,\frac{1}{2}\right)$. Thus, segment AB can be expressed as

(47) $$\begin{array}{c} g=-\frac{19\sqrt{2}}{36}d_{w}+\frac{1}{2}. \end{array}$$

### 5.2. Segment II

The entanglement witness matrix of this segment is similar to that of Segment I. The difference is $\omega_{3}$, and it is represented as follows:

(48) $$\omega_{3}=u\left[\begin{array}{cc} \frac{1}{h} & 1\\ 1 & h \end{array}\right],$$

where *h* and *u* are positive parameters. Similar to the process of Segment I, the denominator in Equation (4) can be represented as

(49) $$\langle \psi_{4}\left|\omega \right|\psi_{4}\rangle =\frac{u}{h}c^{8}+2uc^{4}s^{4}+hus^{8}+6\left(d+6e+f\right)c^{4}s^{4}+4\left(4b+a\right)c^{6}s^{2}.$$

According to the analytical calculation shown in Table 1, we have obtained that in this part, there are two θ that simultaneously cause Equation (49) to reach the maximum value, one of which is θ=π, and the maximum is hu. Substituting the maximum value hu into Equation (49), we can easily obtain

(50) $$\frac{1}{h^{2}}c^{8}+2\frac{1}{h}c^{4}s^{4}+s^{8}+\frac{6\left(d+6e+f\right)c^{4}s^{4}+4\left(4b+a\right)c^{6}s^{2}}{hu}-1=0.$$

Apart from θ=π, we substitute $\theta =78.4630^{\circ }$ into the formula and can easily get the relationship between other parameters and *h*. The numerator in Equation (4) can be expressed as

(51) $$\langle \rho ,\omega \rangle =\frac{1-p}{2}\left[\left(d+6e+f\right)+\left(4a+b\right)\right]+\frac{p}{2}\left(\frac{u}{h}+2u+hu\right).$$

According to the conditions for obtaining the maximum value, we get the relationship between *h* and Θ as

(52) $$h=\frac{\frac{\tan\Theta }{\sqrt{2}}-\frac{1}{6}}{-\frac{\tan\Theta }{\sqrt{2}}+\frac{3}{8}},$$

where $\tan\Theta =\frac{p}{\frac{1}{\sqrt{2}}\left(1-p\right)}$. Substituting Equation (49), Equation (51), and the relationship between parameters into Equation (4), we get the lower bound of the robustness of entanglement in this segment:

(53) $$g=\frac{\sin\Theta }{R(\sin\Theta +\sqrt{2}\cos\Theta )}=\frac{2x}{\frac{{\left(x-\frac{1}{6}\right)}^{2}}{\frac{3}{8}-x}+x+\frac{203}{72}},$$

(54) $$d_{w}=\frac{\sqrt{2}\cos\Theta }{R(\sin\Theta +\sqrt{2}\cos\Theta )}=\frac{1}{\frac{{\left(x-\frac{1}{6}\right)}^{2}}{\frac{3}{8}-x}+x+\frac{203}{72}},$$

where $x=\frac{1}{\sqrt{2}}\tan\Theta$.

The upper bound of this segment is shown in Figure 3 with a blue line.

### 5.3. Segment III

The entanglement witness matrix of this segment is similar to that of Segment II. The difference is $\omega_{3}$, and it is represented as follows:

(55) $$\omega_{3}=u\left[\begin{array}{cc} 0 & 0\\ 0 & h \end{array}\right],$$

where *h* is a positive parameter. Similar to the process of Segment I, the denominator in Equation (4) can be represented as

(56) $$\langle \psi_{4}\left|\omega \right|\psi_{4}\rangle =hs^{8}+6\left(d+6e+f\right)c^{4}s^{4}+4\left(4b+a\right)c^{6}s^{2}.$$

According to the analytical calculation shown in Table 1, we have determined that in this part, there are two θ that simultaneously cause Equation (56) to reach the maximum value, one of which is θ=π, and the maximum is *h*. Substituting the maximum value *h* into Equation (56), we can easily obtain

(57) $$s^{8}+\frac{6\left(d+6e+f\right)c^{4}s^{4}+4\left(4b+a\right)c^{6}s^{2}}{h}-1=0.$$

Apart from θ=π, we substitute $\theta =78.4630^{\circ }$ into the formula and can easily get the relationship between other parameters and *h*, and it is expressed as follows:

(58) $$\frac{16}{625}+\frac{1}{h}\frac{216}{625}\left(A+B\right)-1=0,$$

where A=d+4e+f, and B=4a+b. The numerator in Equation (4) can be expressed as

(59) $$\langle \rho ,\omega \rangle =\frac{1-p_{1}}{2}\left[p_{2}A+\left(1-p_{2}\right)B\right]+\frac{p_{1}}{2}h.$$

Substituting Equation (56), Equation (59), and the relationship between parameters analyzed above into Equation (4), we get the lower bound result of the robustness of entanglement in this segment:

(60) $$\frac{203}{72\sqrt{2}}d_{w}+\frac{1}{2}g=1.$$

Next, we will present the upper bound of this segment. $\sigma_{D}$ corresponding to point *D* can be expressed as

(61) $$\begin{array}{cc} \sigma_{D}= & \frac{216}{625}(|W\rangle \langle W|+|Dicke\rangle \langle Dicke|)+\frac{32}{625}\left|GHZ\rangle \langle GHZ\right|+\frac{377}{625}\tau , \end{array}$$

where τ is the noise state.

The expression of the separable state $\sigma_{C}$ contains a higher proportion of |GHZ〉 than $\sigma_{D}$. Based on Equation (45) and $\sigma_{D}$, we still have $\frac{20}{625}$ of |0000〉〈1111|+|1111〉〈0000| that can be transformed into GHZ states. By adding $\frac{20}{625}\left|1111\rangle \langle 1111\right|$ and normalizing, which is give by $\sigma_{C}=\frac{1}{1+p}(\sigma_{D}+p|1111\rangle \langle 1111|)$, the expression of $\sigma_{C}$ is obtained as follows:

(62) $$\begin{array}{cc} \sigma_{C}= & \frac{24}{215}\left|GHZ\rangle \langle GHZ\right|+\frac{72}{215}(|W\rangle \langle W|+|Dicke\rangle \langle Dicke|)\\ & +\frac{32}{215}|\bar{W}\rangle \langle \bar{W}|+\frac{15}{215}|0000\rangle \langle 0000|. \end{array}$$

Therefore, the point *C* is $\left(\frac{72}{215}\sqrt{2},\frac{24}{215}\right)$ with $\Theta =13.26^{\circ }$. Similarly, $\sigma_{CD}=\left(1-p\right)\sigma_{C}+p\sigma_{D}$, the expression of segment CD can be represented as

(63) $$\begin{array}{c} g=-\frac{203\sqrt{2}}{72}d_{w}+2. \end{array}$$

### 5.4. Segment IV

The entanglement witness matrix of this segment only has two parts: $\omega_{1}$ and $\omega_{2}$. The denominator in Equation (4) can be represented as

(64) $$\langle \psi_{4}\left|\omega \right|\psi_{4}\rangle =6\left(d+6e+f\right)c^{4}s^{4}+4\left(4b+a\right)c^{6}s^{2}.$$

According to the analytical calculation shown in Table 1, we determine that in this part, there is only one θ that causes Equation (64) to reach the maximum value, and we can easily obtain

(65) $$\langle \sigma ,\omega \rangle =\frac{216}{625}\left[\left(d+6e+f\right)+\left(4b+a\right)\right].$$

The numerator in Equation (4) can be expressed as

(66) $$\langle \rho ,\omega \rangle =\frac{1-p_{1}}{2}\left[p_{2}\left(d+6e+f\right)+\left(1-p_{2}\right)\left(4b+a\right)\right].$$

Combining the above analysis structures, we get the lower bound result of robustness of entanglement in this segment:

(67) $$d_{w}=\frac{216}{625}\sqrt{2}.$$

Therefore, $\sigma_{E}$ corresponding to point *E* can be expressed as

(68) $$\begin{array}{c} \sigma_{E}=\frac{216}{625}(|W\rangle \langle W|+|Dicke\rangle \langle Dicke|)+\frac{409}{625}\tau , \end{array}$$

where τ is the noise state. It is evident that $w=d=\frac{216}{625}$ for segment DE, and *g* increases from 0 to $\frac{32}{625}$ as Θ increases. Therefore, *D* is $\left(\frac{216}{625}\sqrt{2},\frac{32}{625}\right)$ with $\Theta =5.98^{\circ }$, and *E* is $\left(\frac{216}{625}\sqrt{2},0\right)$ with $\Theta =0^{\circ }$. The separable state $\sigma_{DE}$ for segment DE can be expressed as $\sigma_{DE}=\left(1-p\right)\sigma_{D}+p\sigma_{E}$, which can represent an arbitrary separable state on the segment DE. By respectively transforming the variables *p* and (1−p) into *g* and $d_{w}$, the expression for the upper bound is derived as follows:

(69) $$\begin{array}{c} d_{w}=\frac{216}{625}\sqrt{2}. \end{array}$$

## 6. Conclusions

In this paper, we propose a method to quantify the multipartite entangled states of quantum resources. We quantify full separability for Dicke–W and GHZ mixed states by analyzing the upper bound and the lower bound of the robustness of entanglement. When Φ is $90^{\circ }$, that is, in the case of Dicke and GHZ mixed states, we obtain the analytical expression of the lower bound and upper bound, and the results show that the lower bound coincides with the upper bound. When Φ is $45^{\circ }$, that is, in the case of equal proportions of Dicke and W states mixed with GHZ state, we obtain analytical solutions for the straight segments and a tractable solution for the curved segment. Finally, we determine that the bound of the Dicke–W mixing ratio is $\Phi =56.31^{\circ }$, which divides the curve of the robustness of entanglement into two types. 

## Figures and Tables

**Table 1:** Segmentation of the four-qubit Dicke–W and GHZ mixed states with a Dicke–W mixing ratio of $\Phi =45^{\circ }$.

Segment Number	Θ	ω	θ	Line Type
Segment I	[20.9576°, 90°]	Type 1	0°, 78.4630°, π	Straight line
Segment II	[13.2627°, 20.9576°]	Type 2	78.4630°, π	Curve
Segment III	[5.9803°, 13.2627°]	Type 3	78.4630°, π	Straight line
Segment IV	[0°, 5.9803°]	Type 4	78.4630°	Straight line

## Data Availability

No new data were created or analyzed in this study. Data sharing is not applicable to this article.

## Appendix A

According to Equations (11) and (12), we get

(A1) $$\begin{array}{cc} \; & Tr(\hat{M^{\prime }}|\psi_{s}\rangle \langle \psi_{s}|)\\ \; & =Tr\sum_{ijkl=0}^{3}M_{ijkl}\sigma_{i}⊗\sigma_{j}⊗\sigma_{k}⊗\sigma_{l}\cdot \rho_{1}⊗\rho_{2}⊗\rho_{3}⊗\rho_{4}\\ \; & =\sum_{ijkl=0}^{3}M_{ijkl}\left[Tr\left(\rho_{1}\sigma_{i}\right)Tr\left(\rho_{2}\sigma_{j}\right)Tr\left(\rho_{3}\sigma_{k}\right)Tr\left(\rho_{4}\sigma_{l}\right)\right]\\ \; & =\Lambda_{1}+\Lambda_{2}x_{4}+\Lambda_{3}y_{4}+\Lambda_{4}z_{4}\\ \; & \leq \Lambda_{1}+\sqrt[{\;}]{\Lambda_{2}^{2}+\Lambda_{3}^{2}+\Lambda_{4}^{2}}, \end{array}$$

(A2) $$\begin{array}{cc} \Lambda_{1}= & M_{2}z_{3}+M_{3}z_{2}+M_{4}z_{1}+M_{5}z_{1}z_{2}z_{3}+M_{6}\left(x_{1}x_{2}+x_{1}x_{3}+x_{2}x_{3}\right.\\ \; & \left.+y_{1}y_{2}+y_{1}y_{3}+y_{2}y_{3}\right)+M_{8}\left(x_{1}x_{2}z_{3}+x_{A}z_{2}x_{3}+z_{1}x_{2}x_{3}\right)\\ \; & +M_{9}\left(y_{1}y_{2}z_{3}+y_{1}z_{2}y_{3}+z_{1}y_{2}y_{3}\right)+M_{11}\left(z_{1}z_{2}+z_{1}z_{3}+z_{2}z_{3}\right),\\ \Lambda_{2}= & M_{6}\left(x_{1}+x_{2}+x_{3}\right)+M_{7}\left(z_{1}z_{2}x_{3}+z_{1}x_{2}z_{3}+x_{1}z_{2}z_{3}\right)\\ \; & +M_{8}\left(x_{1}z_{2}+x_{1}z_{3}+x_{2}z_{3}+z_{1}x_{2}+z_{1}x_{3}+z_{2}x_{3}\right)\\ \; & +M_{12}\left(x_{1}y_{2}y_{3}+y_{1}x_{2}y_{3}+y_{1}y_{2}x_{3}\right)+M_{13}x_{1}x_{2}x_{3}\\ \Lambda_{3}= & M_{6}\left(y_{1}+y_{2}+y_{3}\right)+M_{7}\left(z_{1}z_{2}y_{3}+z_{1}y_{2}z_{3}+y_{1}z_{2}z_{3}\right)\\ \; & +M_{9}\left(y_{1}z_{2}+y_{1}z_{3}+y_{2}z_{3}+z_{1}y_{2}+z_{1}y_{3}+z_{2}y_{3}\right)\\ \; & +M_{12}\left(x_{1}x_{2}y_{3}+x_{1}y_{2}x_{3}+y_{1}x_{2}x_{3}\right)+M_{14}y_{1}y_{2}y_{3},\\ \Lambda_{4}= & M_{1}+M_{5}\left(z_{1}z_{2}+z_{1}z_{3}+z_{2}z_{3}\right)+M_{7}\left(z_{1}x_{2}x_{3}+x_{1}z_{2}x_{3}\right.\\ \; & \left.+x_{1}x_{2}z_{3}+z_{1}y_{2}y_{3}+y_{1}z_{2}y_{3}+y_{1}y_{2}z_{3}\right)+M_{8}\left(x_{1}x_{2}+x_{1}x_{3}\right.\\ \; & \left.+x_{2}x_{3}\right)+M_{9}\left(y_{1}y_{2}+y_{1}y_{3}+y_{2}y_{3}\right)+M_{10}z_{1}z_{2}z_{3}\\ \; & +M_{11}\left(z_{1}+z_{2}+z_{3}\right). \end{array}$$

Thus, we get Equation (13).
